# A new paradigm for tumour profiling: Spatiotemporal omics in living tissue

**DOI:** 10.1002/ctm2.70547

**Published:** 2025-12-08

**Authors:** Ciro Chiappini

**Affiliations:** ^1^ Centre for Craniofacial and Regenerative Biology, Faculty of Dentistry, Oral and Craniofacial Sciences King's College London London UK; ^2^ London Centre for Nanotechnology King's College London London UK

## 1 DYNAMIC TUMOUR BIOLOGY DRIVES THERAPEUTIC RESPONSE

One of the central challenges in oncology is understanding why tumours stop responding to therapy. Clinicians see this repeatedly: an initial response that gives way to relapse, often driven by the tumour's ability to adapt at the molecular level. These adaptations are not static. They unfold over hours, days, and weeks, and they vary across different regions of the same tumour.

**FIGURE 1:**
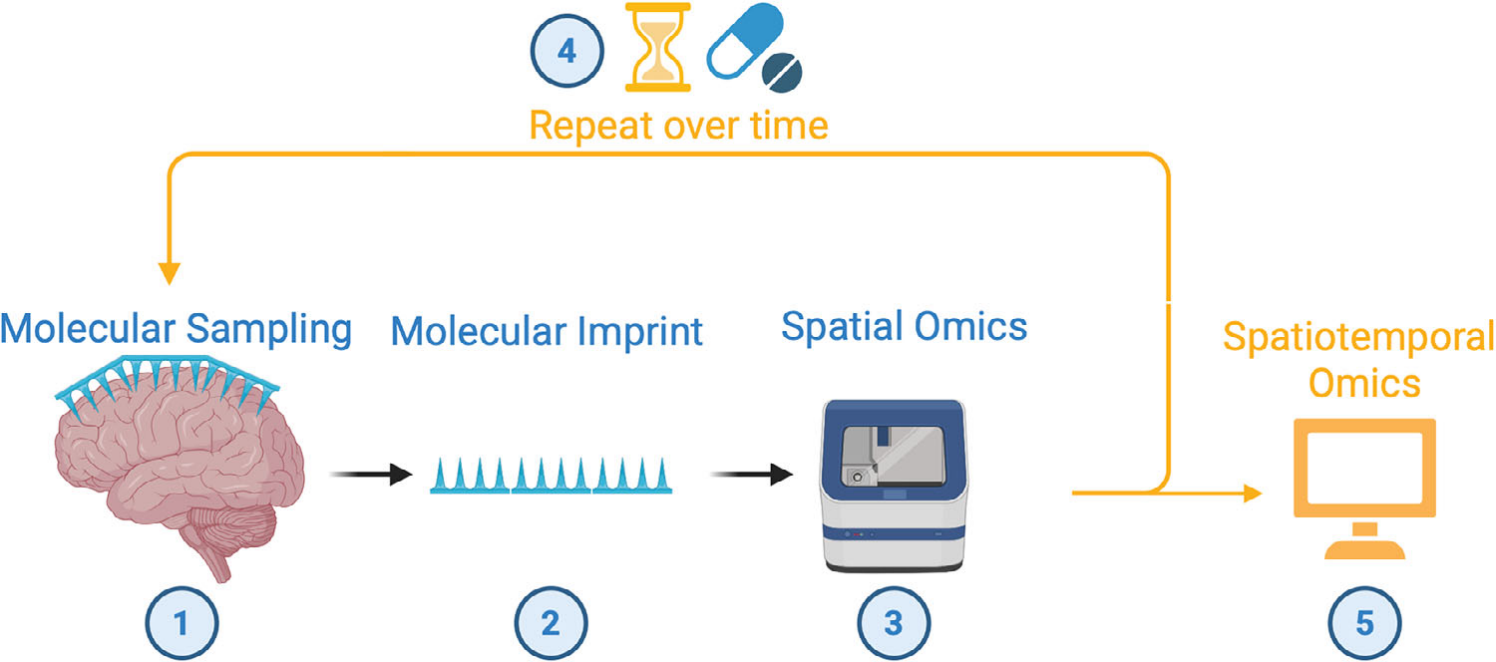
A new paradigm for molecular profiling. (1) Nondisruptive nanoneedle sampling of living tissue generates (2) molecular imprints that preserve spatial organisation and molecular composition. (3) These imprints support spatial omics analysis, and the nondisruptive sampling enables (4) repeated profiling the same tissue during treatment. (5) This approach enables spatiotemporal molecular profiling in living tissue, offering a means to study emerging resistance dynamics relevant to precision intervention. Created in BioRender. Chiappini (2025). https://BioRender.com/bzj0gpq.

This means that the molecular programmes that enable a tumour to escape treatment are *dynamic, spatially organised, and highly patient‐specific*. Yet the tools we use today, bulk sequencing, fixed‐tissue analysis, and endpoint assays, capture only isolated moments in time. They fall short when clinicians need to know how a tumour changes during treatment, where resistance emerges within the tissue, and when a vulnerable state might be present. This gap calls for a new paradigm for tumour profiling: capturing molecular dynamics directly in living tissue, in both space and time (Figure 1).

## 2 CAPTURING DYNAMIC RESISTANCE WITH SPATIOTEMPORAL OMICS

Emerging spatial and temporal omics technologies are heralding this paradigm, but each captures only part of the picture.

Spatial omics platforms provide detailed maps of fixed biopsy material, yet remain static snapshots that cannot show how these patterns change once treatment begins. Temporal profiling methods offer insight into dynamic responses but rely on sequential biopsies and cannot reveal where within the tissue those changes arise. Lineage tracing,^1^ metabolic labelling,^2^ and live‐tissue imaging^3^ each contribute fragments of the picture, but they either require destructive processing, genetic manipulation, or provide only limited molecular depth.

The core challenge remains: we can map a tumour's landscape or track its evolution, but not capture both in living tissue. This limits our ability to detect early resistance and make timely, biology‐guided decisions.

## 3 NANOTECHNOLOGY FOR LIVE MOLECULAR PROFILING

A key barrier in studying treatment response is that most molecular analyses require destroying the tissue. This makes it impossible to follow how the same piece of patient‐derived material changes over time. Nanotechnology now offers a way around this by enabling longitudinal molecular sampling: the ability to extract small amounts of intracellular material from living tissue without compromising its viability.

Pioneering work using single‐probe technologies such as nanopipettes^4^ and FluidFM^5^ showed that it is possible to take ‘live‐cell biopsies’: tiny samples of RNA, proteins, or metabolites from the same living cell at multiple timepoints. These studies proved the concept that molecular pathways can be monitored dynamically in living systems, a breakthrough step for temporal omics. However, these approaches work cell‐by‐cell and are not scalable to tissue‐level analysis or to most types of patient‐derived samples used in clinical research.

Nanoneedle arrays overcome these practical limitations.^6^ Rather than sampling one cell at a time, arrays of thousands of microscopic needles can interface with many cells simultaneously across a tissue.^7^ They gently access the cytoplasm, withdraw minimal material, and leave the cells intact.^8^ Importantly, this can be repeated allowing clinicians and researchers to follow the same tissue as it adapts to treatment, without the need for repeat biopsies or destructive processing.

Through this nondisruptive sampling, nanoneedles recover RNA, proteins, and metabolites at yields compatible with modern omics analyses while preserving tissue structure and function. This creates, for the first time, a platform for *spatiotemporalmulti‐omics profiling of living human tissue*.

## 4 CAPTURING TREATMENT RESPONSE WITH NANONEEDLES

**FIGURE 2:**
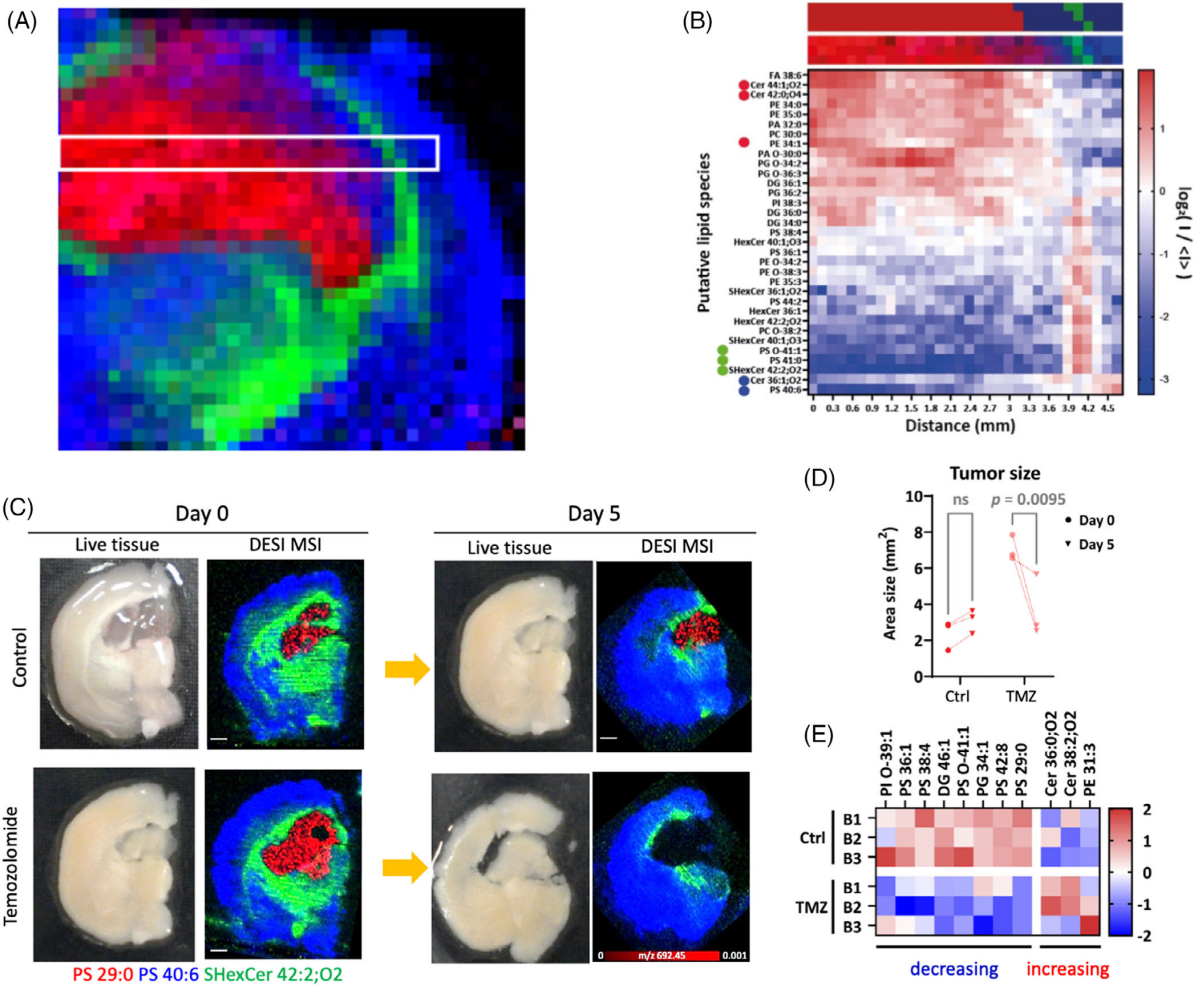
Revealing metabolic adaptation to therapy in living tissue. (A) Spatial lipidomic map of a nanoneedle imprint from a glioma‐bearing mouse brain, highlighting tumour (red), white matter (green), and grey matter (blue). (B) Heatmap showing the distribution of the most characteristic lipids within the boxed region in (A), illustrating distinct lipidomic signatures of tumour, white matter, and grey matter. (C) Spatiotemporal lipidomic maps of glioma tissue before and after temozolomide treatment, showing treatment‐associated loss of phosphatidylserine (PS 29:0). (D) Quantification of tumour size over time derived from the spatiotemporal lipidomic maps. (E) Heatmap of treatment‐induced changes in tumour lipid composition. Adapted from Ref (10).

In our recent study, we applied this technology to living human glioma tissue and demonstrated what has long been needed in clinical oncology: the ability to track how a patient's tumour responds to *treatment in both space and time* ^9^ (Figure 2).

Nanoneedle sampling produced a ‘molecular imprint’ of the tissue, capturing the spatial organisation of metabolites with high fidelity, while keeping the tissue alive. These imprints preserved the molecular patterns that distinguish high‐grade from low‐grade glioma, and they identified the same biomarker signatures that would ordinarily require destructive analysis of the original tissue. Crucially, because the process is nondisruptive, we could sample the *same* tissue before and after exposure to chemotherapy.

This allowed us, for the first time, to directly observe the *spatiotemporal metabolic response* of a living tumour to treatment. We could see where in the tissue metabolic signatures were rewired, how the abundance of key lipids shifted, and how these changes unfolded over time. This direct measurement of treatment‐induced dynamics opens the door to understanding resistance as it emerges in real time.

## 5 CLINICAL OPPORTUNITIES

Our ability to profile living tissue in both space and time creates new opportunities across clinical research, precision oncology, and patient care. For clinicians, the most immediate value lies in understanding how an individual patient's tumour responds to therapy before those changes manifest radiologically or clinically.

In precision medicine and translational research, nanoneedle sampling enables dynamic testing of therapeutic responses directly on living patient‐derived tissue. By capturing early molecular shifts that indicate sensitivity or emerging resistance, it helps clinicians choose effective drug combinations and avoid regimens vulnerable to adaptive escape. Longitudinal imprints simultaneously give researchers early efficacy signals, clarify heterogeneous responses across cohorts, and show how candidate therapies reshape key molecular pathways.

Minimally invasive longitudinal sampling has the potential to reduce the burden of repeat biopsies. We have developed ways to integrate nanoneedles into familiar clinical instruments,^10^ such as patches, bandages, endoscopes, catheters, and angioplasty balloons, allowing clinicians to obtain molecular imprints from accessible lesions without removing tissue. Repeated sampling of precancerous or inflammatory lesions during surveillance programmes could support early detection of progression and guide timely intervention.

Intraoperatively, molecular imprints could offer more informative feedback than frozen section analysis, without the need for tissue excision. Their additional molecular depth may strengthen decision‐making, particularly for margin assessment and treatment escalation.

Together, these capabilities point to a future where clinicians can follow a patient's disease biology in real time, inform treatment selection with direct evidence of dynamic molecular response, and reduce the procedural burden associated with traditional tissue sampling. Nanoneedle‐enabled spatiotemporal profiling thus represents a promising step toward more adaptive, personalised, and clinically actionable molecular medicine.
